# Supplementary material for: Stepwise on-surface synthesis of nitrogen-doped porous carbon nanoribbons
Source: Commun Chem. 2024 Feb 24;7:40. doi: 10.1038/s42004-024-01123-4 (PMC10894233; doi:10.1038/s42004-024-01123-4)
Supplement: Supplementary file 2 — Supplementary Information [file 42004_2024_1123_MOESM2_ESM.pdf]

Supplementary Information
for

Stepwise on-surface synthesis of nitrogen-doped porous carbon nanoribbons

Jin Xu,[1] Shuaipeng Xing,[1] Jun Hu*[2], Ziliang Shi*[1]

[1] Center for Soft Condensed Matter Physics & Interdisciplinary Research, School of Physical Science and Technology, Soochow University, Suzhou 215006, China. phzshi@suda.edu.cn
[2] School of Physical Science and Technology, Ningbo University, Ningbo 315112, China. hujun2@nbu.edu.cn

Table of Contents

1. The length distributions of ZZC polymers
2. Vibration of individual ZZCs
3. Additional STM data
4. The histograms of the length of n-NPCNs
5. DFT results of pure porous graphene
6. Band structures of n-NPCNs

1. The length distributions of ZZC polymers

To test the substrate effect on the intrinsic selectivity in molecular conformation and intermolecular debromination, we have analyzed the length distributions of ZZC polymers on different substrates. The total number of ZZCs is 84, 152 and 214 for the Au, Ag and Cu samples, respectively. Figure S1 shows the length distributions of the three samples. The average length is 31 nm, 26 nm and 17 nm for the ZZC polymers on Au(111), Ag(111) and Cu(111), respectively. We tentatively attribute the non-chain products on Cu(111) to the Cu adatoms affording a metal-coordination effect, which induces the *trans–cis* conformational transition of DT monomers and the emergence of *cis* covalent coupling modes.

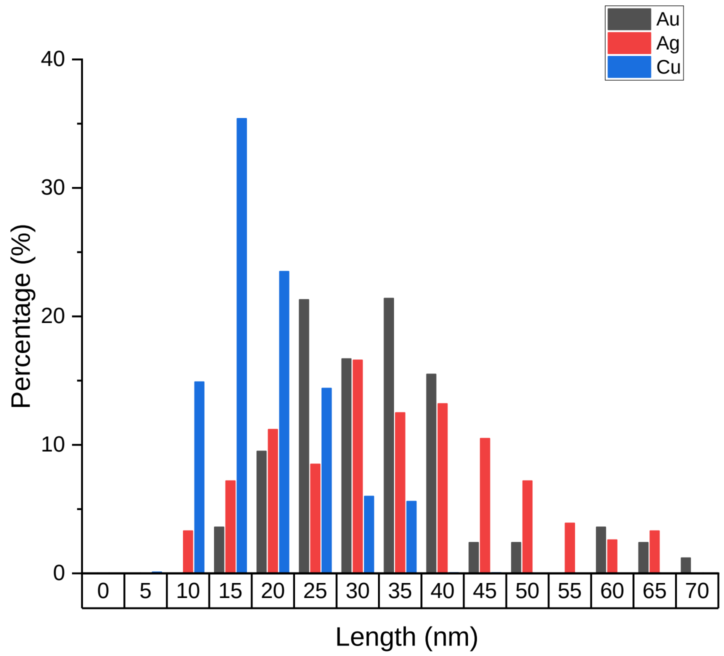

Figure S1. The length distributions of ZZCs on Au(111) (green), Ag(111) (red) and Cu(111) (blue).

2. Vibration of individual ZZCs

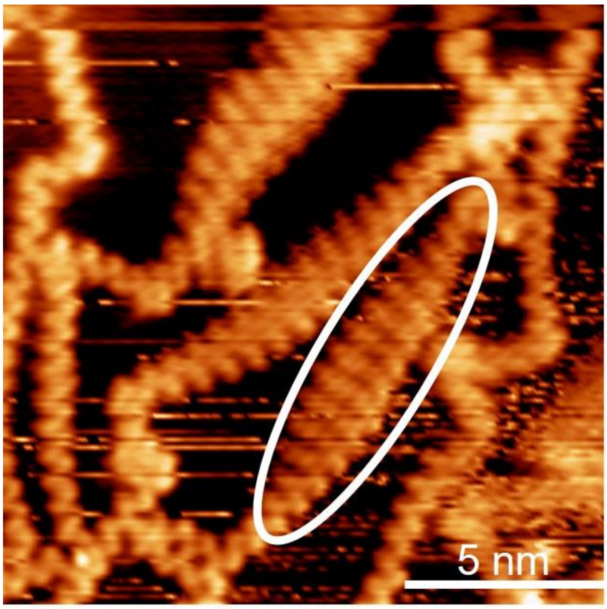

Figure S2. The vibrational motion of individual ZZCs on Au(111). Data acquisition conditions: U = -1.1 V, I = 0.5 nA; The STM image was acquired at room temperature.

3. Additional STM data

To demonstrate the metal-coordination effect of Cu adatoms, we have deposited DT onto a Au(111) substrate preadsorbed with Cu; see Cu islands on a clean Au(111) surface in Fig. S3a. An annealing at 370 K steered the intermolecular debrominative coupling, which created meandering polymeric structures; see the STM overview in Fig. S3b and a close inspection in Fig. S3c. The products, dramatically distinct from that in the absence of Cu adatoms, include short ZZC segments, foldings, kinks, and even rings. We therefore conclude that the formation of Cu-N coordination induces the change in molecular conformation and in intermolecular coupling modes, and thus suppresses the intrinsic selectivity of the on-surface reaction.

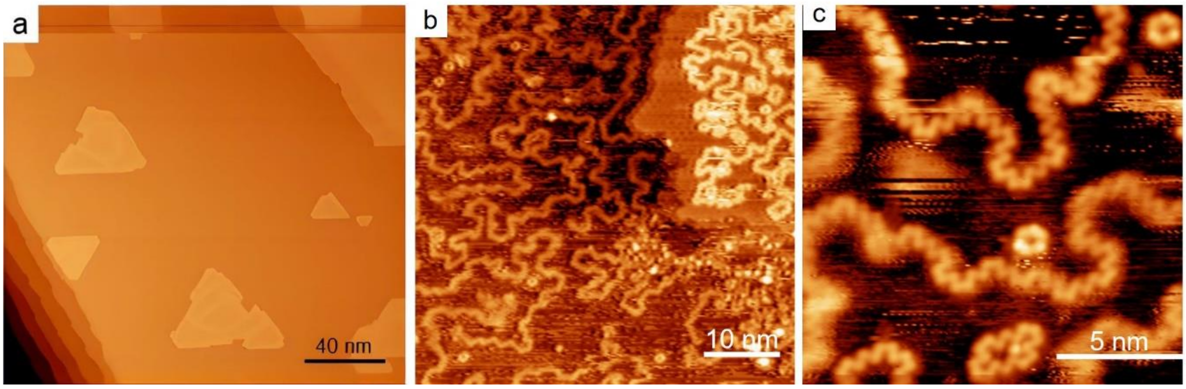

Figure S3. (a) Cu islands on Au(111). (b) Polymeric structures after the intermolecular debrominative coupling following an annealing at 370 K. (c) Close inspection of the polymeric structures. Data acquisition conditions: (a) U = -1.2 V, I = 50 pA ; (b) U = -1.0 V, I = 50 pA; (c) U = -0.8 V, I = 100 pA. All STM images were acquired at room temperature.

4. The histograms of the length of n-NPCNs

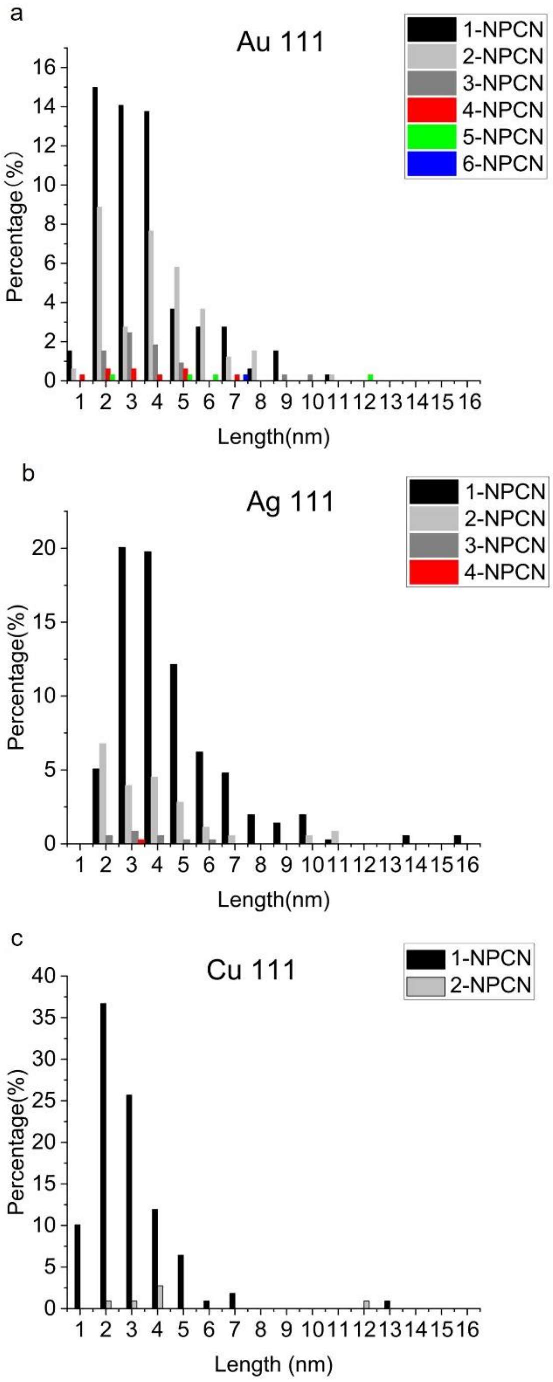

Figure S4. The histograms of the length of n-NPCNs on (a) Au(111), (b) Ag(111) and (c) Cu(111). The total number of chains is 323, 354 and 109 for the Au, Ag and Cu substrate, respectively. Color: 1-NPCN, black; 2-NPCN, light gray; 3-NPCN, gray; 4-NPCN, red; 5-NPCN, green; 6-NPCN, blue.

5. DFT results of pure porous graphene

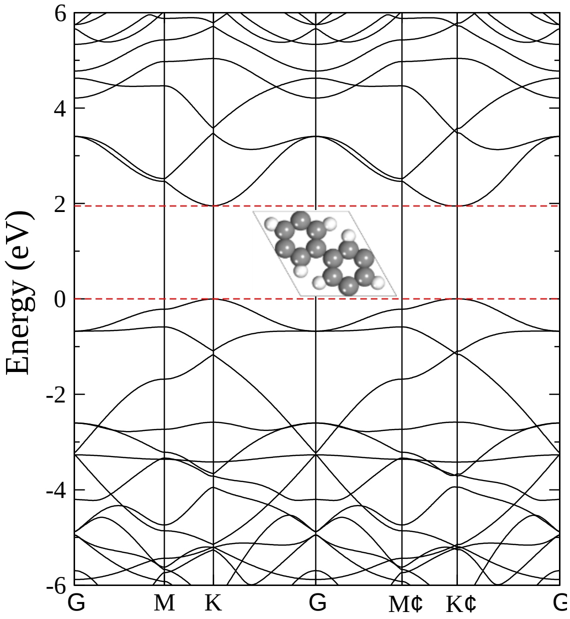

Figure S5. Band structures of pure porous graphene. The horizontal dashed lines indicate the GGA-PEB band gap. The inset shows the unit cell.

6. Additional DFT results

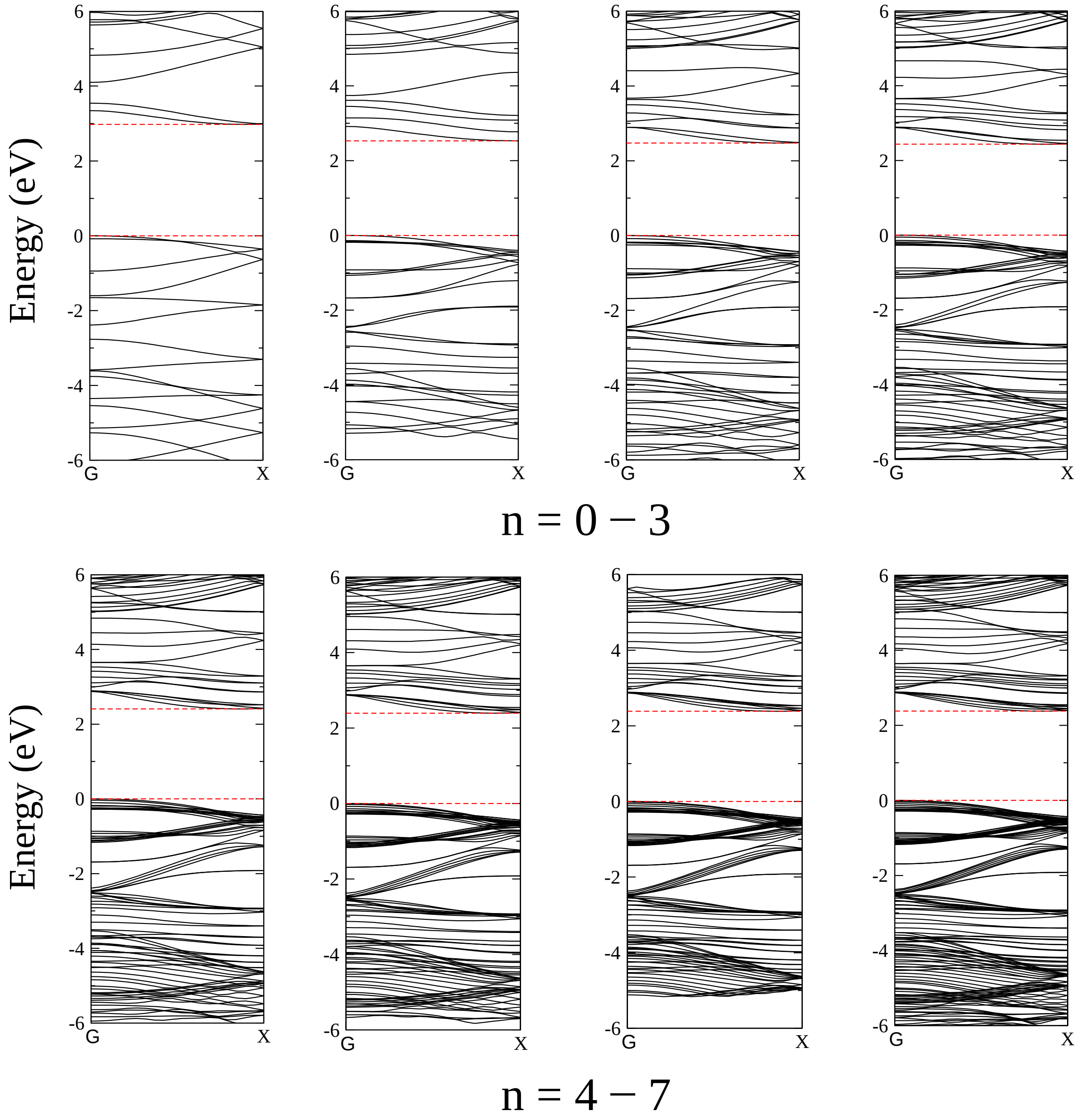

Figure S6. Band structures of n-NPCNs, n=0-7.
